# Microscopical Memoranda

**Published:** 1874-05-15

**Authors:** 


### Microscopical Memoranda.

COLLATED BY LESTER CURTIS, M.D.

ON THE LEUCOCYTE AND PUS CORPUSCLE.—The *Philadelphia Medical Times* of March 28th contains a report of the proceedings of the Philadelphia Pathological Society, from which we quote the following:

"Dr. Bertolet said he had not a clear idea of what was comprised by the term "*leucocytes*," and desired much to know its limitations.

"Dr. Tyson replied that, after other better-known histologists, he had always used the word *leucocyte* in a generic sense, as including all that class of small, round, variously granular cells which, according to the situations in which they were found, were variously called white blood-corpuscles, mucus-corpuscles, young pus-corpuscles, or the round cells of connective tissue,—in other words, *dead amœboid cells.*

"Dr. Bertolet said he thought this was an error in theory which had been allowed to supplant practice; that the white corpuscle and pus-corpuscle were not the same.

"Dr. Richardson said the word *leucocyte* had been originally introduced by Charles Robin, who applied it to the class of bodies named by Dr. Tyson, whether alive or dead, as well as to exudation-corpuscles, and he believed also, provisionally, salivary corpuscles. He thought that if any one would treat white corpuscles contained in a drop of blood from his finger, first with water by introducing a small quantity at the edge of the thin glass cover, and then with weak aniline solution, in the manner described in his report on the white blood-corpuscle (Amer. Med. Assoc. Trans., 1872), he would have no difficulty in finding many globules which exhibited two or three, and occasionally those which displayed four or five, well-formed and strongly-tinted nuclei, and which manifested a precise identity, in that respect at least, with the leucocytes of pus, as described by older pathologists. By this experiment it was easily demonstrated that the characteristic formerly so much relied upon for the recognition of the pus-cell, and quoted by Dr. Bertolet,—namely, that it possessed two, three, or more nuclei,—was valueless as a means for its discrimination from the leucocytes of blood.

"Dr. Tyson admitted that pus-corpuscles soon became very granular from fatty degeneration, and then presented objects which did not so closely resemble the white blood-corpuscle; but in their young state he did not think they could be distinguished, and to acetic acid and water both responded identically.

"Dr. Richardson said that about one white corpuscle out of thirty is ordinarily more granular than its companions, and he was strongly inclined to think that these white corpuscles were also the seat of fatty degeneration.

"The President said it was very important to have clear ideas as to the exact application of terms. He presumed, of course, that this discussion referred simply to the morphology, and not the vital properties or developmental tendencies, of the cells in question. He said that he himself had been called upon to study cases where inflammation had obliterated the trunks of vessels,—a matter which brought up directly the question of being able to distinguish between the corpuscles in the surrounding inflamed tissue and the white corpuscles which remained in the softened clots. By no means which were available could he distinguish between the two.

"Dr. Richardson thought the more he studied the subject in connection with Cohnheim's observations, the more he was led to conclude that *living leucocytes of pus and blood were identical physiologically as well as morphologically.*"

The italics are our own.

---

BLUE PUS.—It is well known that pus, and the dressings of neglected wounds, sometimes show a blue color. The *American Journal of the Medical Sciences* contains an article on this subject condensed from the *Medical Times and Gazette*, and one from the *Archives Gen. de Med.* This blue color of pus may become epidemic, as was the case in M. Gosselin's wards in the Charite at Paris. Cases are also on record in which the normal secretions, as sweat, milk, and urine, have been of this color. Two sources for the color are indicated: one, the hæmoglobin; the other, the indican of the urine. Hæmoglobin, effused, assumes the varying colors seen in a bruise; in an old clot it becomes hæmatoidin, identical with the red coloring matter of the bile. The action of nitric acid, which is a process of oxidation, produces a blue tint in the bile. Perhaps a similar oxidation of hæmoglobin gives rise to the blue of the secretion, or the pus.

Various theories have been advanced to explain the blue coloring of the dressing of wounds. One, favored by Lucke, is that it is due to vibrios. [Our readers will bear in mind the theory that all decomposition is caused by fungi, or other microscopic organisms.] This theory of the vibrios would explain the epidemics, if we might so call them, which occur where there is a neglect of cleanliness, and in hot, moist weather.

A blue coloring matter, called pyocyanine, has been isolated from blue pus, which resembles indican, a blue coloring matter, occurring as a normal constituent of urine, and probably of the blood also. This indican may be the source of the blue color of the pus and secretion.

*The Archives de Med.* considers the blue color to be of three kinds: 1st. The coloration resulting from the modification of certain humors—or true blue coloration; 2d. Coloration due to fungi—false blue coloration; 3d. Coloration due to a substance still unknown—false blue suppuration. The name *cyanchrose* is proposed for the last. It commences and ceases suddenly, not only where there is a wound, but where the parts are sound. Its duration is variable. It produces no modification in the local condition of the wound, or the general health of the patient. Its progress is similar to that of erysipelas. It is sometimes epidemic. It occurs most frequently when the atmosphere is moist and warm and contains ozone, and during storms. Its presence is a favorable prognostic.

---

ORIGIN OF PUS CORPUSCLES IN INFLAMED CORNEA.—Prof. A. Bottcher contributes a paper on this subject to *Virchow's Archiv.* It is well known that Conheim maintains that traumatic keratitis always begins at the periphery of the cornea, and extends inwards, and that the corneal corpuscles are unaltered; in other words, that the pus corpuscles in keratitis come from the white corpuscles of the blood. Bottcher, after numerous experiments, obtains results differing from this. By touching the cornea with a point of chloride of zinc, moderated with nitrate of potash, he is able to produce a limited keratitis without any hazy zone at the margin, and has found, also, that the corneal corpuscles are always modified.—*Lancet.*

---

HISTOLOGY OF THE SUDORIPAROUS GLANDS. — M. Krause (*Centralblatt*, No. 52, 1873) states that the sudoriparous glands of the palm and scalp have a single layer of columnar epithelium lining them throughout. To show this, he treats the skin successively with chromic acid, alcohol, and hæmatoxylin; and then makes sections. His hæmatoxylin is composed of decoction of logwood, 30 to 60 parts; alum, 5; chromate of potash, 0.1; creosote, 0.2; filter. He mounts his sections by using, first, absolute alcohol, then oil of cloves, then Canada balsam dissolved in chloroform. —*Lancet.*

---

A STORY is related of a Chicago physician, who is also an extensive real estate operator, that recently he prescribed some pills for a lady, at a time when he was very much absorbed in one of his land transactions. She asked how they were to be taken. "A quarter down," said the doctor, "and the balance in one, two and three years."—*Philadelphia Med. aud Surg. Reporter.*

